# A roadmap toward implementing health technology assessment in Egypt

**DOI:** 10.3389/fpubh.2022.896175

**Published:** 2022-12-13

**Authors:** Ahmad Nader Fasseeh, Baher Elezbawy, Mary Gamal, Ahmed Seyam, Asmaa Abourawash, Mohsen George, Mohamed Anwar, Magdy Amin, Ahmed Yehia Khalifa, Amr Elshalakani, Ashraf Hatem, Sohir Abdelhamid, Hossam Elsamouly, Nader Fasseeh, Randa Adel, Hatem Dawood, Sherif Abaza, Zoltán Kaló

**Affiliations:** ^1^Syreon Middle East, Alexandria, Egypt; ^2^Faculty of Social Sciences, Eötvös Loránd University, Budapest, Hungary; ^3^Egyptian Authority for Unified Procurement, Medical Supply and Technology Management, Cairo, Egypt; ^4^Universal Health Insurance Authority, Cairo, Egypt; ^5^Egyptian Drug Authority, Cairo, Egypt; ^6^Health Insurance Organization, Cairo, Egypt; ^7^Department of Data Management and Decision Support, General Authority of Healthcare, Cairo, Egypt; ^8^Department of Surgery, Military Medical Academy, Cairo, Egypt; ^9^World Health Organization Representative Office, Cairo, Egypt; ^10^Health, Nutrition and Population Global Practice, World Bank, Cairo, Egypt; ^11^Faculty of Medicine, Cairo University, Cairo, Egypt; ^12^American University in Cairo, Cairo, Egypt; ^13^Egyptian Parliament Health Sector, Cairo, Egypt; ^14^Pharmaco-Economics Unit, Police Hospitals, Cairo, Egypt; ^15^Faculty of Medicine, Alexandria University, Alexandria, Egypt; ^16^Takeda Pharmaceuticals, Cairo, Egypt; ^17^Janssen Pharmaceutical Companies of Johnson & Johnson, Cairo, Egypt; ^18^Syreon Middle East, Cairo, Egypt; ^19^Center for Health Technology Assessment, Semmelweis University, Budapest, Hungary; ^20^Syreon Research Institute, Budapest, Hungary

**Keywords:** health technology assessment, HTA, Egypt, health policy, HTA implementation, healthcare system

## Abstract

**Background:**

The Egyptian healthcare system is currently in the early phase of health technology assessment (HTA) implementation. The aim of this study is to propose an implementation roadmap based on the national healthcare system status.

**Methods:**

A survey was conducted among Egyptian healthcare sector decision-makers to assess the current and future (preferred) HTA implementation status in Egypt based on a widely used international scorecard methodology. Subsequently, interviews were conducted with experts representing middle- and top-tier management in the Egyptian healthcare system to interpret the survey results and recommend specific actions.

**Results:**

Experts recommended more capacity-building programs for HTA and health economics. Additionally, they proposed establishing HTA units in separate healthcare authorities and merging them into a single central HTA unit in the long term. Regarding the scope of implementation, experts recommended commencing with the assessment of innovative pharmaceuticals, and thereafter, expanding the scope to cover all health technologies in the long term. Additionally, they recommended using innovative tools such as “multi-criteria decision analysis (MCDA)” for tendering, and “managed entry agreements” for reimbursement decisions. Local burden of diseases and costing studies were also recommended to facilitate the implementation of HTA.

**Conclusion:**

Experts agreed that several actions are required for successful HTA implementation in Egypt, including coordination between HTA bodies, application of an explicit MCDA framework, and strengthening of local evidence generation. To implement these actions, investment in technical capacity-building is indispensable. Most experts favored using multiple and soft cost-effectiveness thresholds. Efforts should be made to publish HTA submission guidelines and timelines of the processes.

## Introduction

Egypt is a lower-middle-income country with a population of approximately 102 million in 2020 (1–3). Its healthcare system is fragmented, with different public and private providers and financing agents. Health services in the country are managed, financed, and provided by agencies in all three sectors of the economy: governmental, parastatal, and private (4).

Egypt has taken several steps to improve its population's health. Political support to increase health spending was obvious in the 2014 constitutional mandate, specifically Article 18, which underlines the right to health and access to good-quality healthcare. Since then, better health information systems have been developed, coverage for health services has improved toward reaching universal health coverage, and more resources have been spent on healthcare (5).

The overall life expectancy increased from 64.5 years to 70.5 years in 2019 (6), reaching 72 years in 2020 (7), approaching the global average of 73 years (8). However, out-of-pocket payments are still considered the major source of healthcare financing in Egypt, accounting for more than 60% of the total healthcare expenditure (9). The risk of catastrophic health expenditure in Egypt is approximately 25%, which is higher than the average for middle-income countries (23%) (7).

Egypt is currently reforming its healthcare system to include universal health insurance (UHI) with broader coverage and better services than the existing insurance system. Upon implementation of the UHI system, its budget should surpass the current total health expenditure (THE), raising THE significantly as a percentage of the gross domestic product (GDP) (9, 10).

Health technology assessment (HTA) refers to the systematic evaluation of the properties, effects, and/or impacts of health technologies. Additionally, it facilitates transparency in decision-making by policymakers based on defined criteria and thresholds (11–15).

Implementing HTA is crucial for achieving the objectives of the UHI system. Primarily, it will enable evidence-based decision-making with clear prioritization and better resource allocation, thus alleviating pressure on the health budget (16, 17). Additionally, it may help expand patients' access to more health services, thereby achieving the broad coverage objective of the UHI. Moreover, it could facilitate controlled diffusion of technologies into the healthcare system. Finally, the formal implementation of HTA has positive spillover effects beyond evidence-based reimbursement decisions (16, 17), such as strengthening dialogue between stakeholders and focusing the national direction toward patient-level outcomes (18).

Although HTA has not yet been fully implemented in Egypt, several steps have been taken toward its implementation. In 2011, the Egyptian chapter of The Professional Society for Health Economics and Outcomes Research (ISPOR) was established, which has provided an impetus to the implementation of HTA in Egypt. In the same year, a ministerial decree was signed to establish the pharmacoeconomics unit (PEU) at the Central Administration of Pharmaceutical Affairs (CAPA). In 2013, the recommendations for reporting pharmacoeconomic evaluations in Egypt were published (19) as the first step toward developing the national guidelines for the economic evaluation of pharmaceuticals. The PEU has received requests for cost-based analyses from various public-sector entities, such as the Tender/Procurement department, Health Insurance Organization, Drug Shortage Department, CAPA pricing committee, and the Ministry of Health technical office.

In 2012, Egypt established the first postgraduate program in health economics; prior to that, only sporadic courses on health economics were provided. Two years later, in 2014, a two-year master's program in health economics started at Cairo University as a joint program between the Faculty of Medicine (Public Health Department) and the Economics Department of the Faculty of Economics and Political Sciences (20, 21). Moreover, a few undergraduate courses have been established, specifically in the faculties of pharmacy in public and private universities (22).

The universal healthcare coverage law, published in 2018, contains a clause for the establishment of the HTA unit within the payer body (23). In 2019, the law for unified procurement was published, directing the establishment of a department for HTA (24).

Additionally, managed entry agreements have been applied in several sectors, and multi-criteria decision analysis (MCDA) tools have been conducted in several tenders by different health authorities. Furthermore, an MCDA tool for purchasing oncology off-patent pharmaceuticals in Egypt was published (25) and operationalized at the Egyptian Authority for Unified Procurement, Medical Supply, and Management of Medical Technology (UPA). The Universal Health Insurance Authority (UHIA) board officially ruled that economic evaluation is a mandatory prerequisite for all new coverage and reimbursement decisions.

Although all the previous steps have helped in HTA implementation, HTA is still not mandatory for pricing or reimbursement at several health authorities. According to the ISPOR Egypt chapter president, the full implementation of HTA in Egypt faces various hurdles. These include the lack of funding and resources for HTA implementation, low quality of available data, and lack of an explicit willingness-to-pay threshold in Egypt (22). Additionally, a gap exists between HTA research and actual reimbursement decision-making (26).

There is a lack of peer-reviewed studies on HTA in Egypt, and no study has yet discussed a systematic plan for implementing HTA in Egypt. However, whatever little literature is available has concurred with the potential benefits of full HTA implementation in Egypt. The purpose of this study is to assess the gaps in HTA implementation in Egypt and to propose a roadmap and specific actions for full HTA implementation over the next decade.

## Materials and methods

Primary and secondary data were used to propose specific actions for HTA implementation in Egypt. First, we evaluated the current status and preferred status of HTA in Egypt through an HTA roadmap survey. Subsequently, interviews were conducted with experts to validate the generated roadmap for feasibility and applicability.

### HTA roadmap survey (HTA scorecard)

The HTA implementation roadmap scorecard/survey developed by Kaló et al. (19) was administered in paper form in July 2018 during a two-day workshop held for Egyptian healthcare decision-makers on HTA implementation. The main purpose of the scorecard was to define the current status of HTA as well as its preferred status in the long run, highlighting the gaps.

The scorecard divides HTA implementation into several domains. For every domain, experts were asked two questions: (1) What is their perception on the current status of HTA regarding this domain? (2) What is their preferred status for that domain in 10 years? Each question had predefined choices for the expert. The distribution of responses reflects the collective opinion of multiple stakeholders, which indicates potential areas for major improvement. As such, the scorecard served as the foundation for advising on the appropriate HTA structure and implementation process from the perspective of key local stakeholders.

The scorecard evaluates eight domains. The first domain, “HTA capacity-building,” is about having well-trained experts for HTA because unfavorable results and system failure may occur if HTA implementation is legislated without adequate capacity. Another domain discusses “Legislation on HTA”—that is, how HTA is integrated into the legal framework. There is a domain about “Scope of HTA implementation,” regarding the range of health technologies that will be evaluated through HTA. “HTA funding” focuses on the HTA system funding model—whether predominantly publicly funded, privately funded, or a mix of both. Other domains discuss “The use of local data,” “Quality and transparency of HTA implementation,” and “Decision criteria”—the latter specifies elements for inclusion in the HTA process. Finally, the “International collaboration” domain assesses international collaboration in the joint assessment of reports and education.

The authors summarized the results of the HTA roadmap survey and constructed draft recommendations based on the major gaps identified through the survey.

### Expert interviews

A series of interviews with stakeholders from different bodies were conducted in June and July 2020 to modulate and validate the draft recommendations. Online interviews were conducted with a diverse group of key stakeholders representing the Egyptian healthcare system's middle- and top-tier management. Interviewees were selected using convenience sampling based on predetermined inclusion criteria: interviewees who had a good understanding of HTA, were influential stakeholders in the Egyptian healthcare system, and represented different public bodies, the private sector, and international organizations were eligible for inclusion. An open-ended structured questionnaire was used for the interviews, which was based on the eight domains of the previous HTA survey results.

At the beginning of each interview, the research objective was introduced to the interviewees, and the progress of the preceding research steps was briefly explained. The interviewer then presented the draft recommendations that were deduced from the survey results. Finally, they were required to comment on the feasibility of implementation, whether they agreed or not on the proposed recommendations, whether they would like to propose any implementation idea/suggestion, and whether they would recommend breaking down the implementation process into phases (short-term to 1–2 years, mid-term to 3–5 years, or long-term to 6–10 years). The interviewees' identities were kept anonymous and only the aggregated results were used for research purposes or publication. We conducted deductive thematic analysis to analyze the responses (27, 28), where the collected data was mapped into the eight HTA domains for creating the final recommendations.

## Results

### General results

#### HTA scorecard survey

Thirty-one local stakeholders completed the HTA scorecard survey, including decision-makers, policymakers, public payers, and potential HTA users. In total, 83.9% indicated working in the public sector, and 16.1% in the private sector. Approximately half of the respondents (48.4%) had training in pharmacy, 22.6% had training in medicine, and 25.8 % were multi-disciplinary—that is, with at least two master's degrees in economics and pharmacy or medicine. Most respondents were in the 30–50 years age group (74.8%). The demographic characteristics are presented in more detail in Supplementary Table 1. The aggregated HTA scorecard results are listed in Table 1. The draft recommendations that were based on the survey responses are summarized in Table 2.

**Table 1:** Aggregated results of valid responses from HTA implementation survey (scorecard).

**Question**	**Current (Current HTA status) *n* (%)**	**Preferred (Aspired situation) *n* (%)**
**1. HTA Capacity-Building**		
**(a) Education**		
No training	6 (19.4%)	0 (0.0%)
Project-based training and short courses	17 (54.8%)	3 (9.7%)
Permanent graduate program with short courses	2 (6.5%)	4 (12.9%)
Permanent graduate and postgraduate program with short courses	6 (19.4%)	24 (77.4%)
**2. HTA Funding**		
**(a) Financing critical appraisal of technology assessment**		
No funding for critical appraisal of technology assessment reports or submissions	27 (87.1%)	1 (3.3%)
Dominantly private funding (e.g., submission fees) by manufacturers for the critical appraisal of technology assessment reports or submissions	4 (12.9%)	14 (46.7%)
Dominantly public funding for critical appraisal of technology assessment reports or submissions	0 (0.0%)	15 (50.0%)
**(b) Financing health technology assessment (i.e., HTA research)**		
No public funding for technology assessment; private funding is not needed or expected	11 (35.5%)	0 (0.0%)
No or marginal public funding for research in HTA; private funding is expected	19 (61.3%)	2 (6.5%)
Sufficient public funding for research in HTA; private funding is also expected	0 (0.0%)	22 (71.0%)
HTA research is dominantly funded from public resources	1 (3.2%)	7 (22.6%)
**3. Legislation on HTA**		
**(a) Legislation on the role of the HTA process and recommendations in the decision-making process**		
No formal role of HTA in decision-making	24 (80.0%)	0 (0.0%)
Dominantly international HTA evidence is taken into account in decision-making	3 (10.0%)	0 (0.0%)
International and additionally local HTA evidence is taken into account in decision-making	3 (10.0%)	20 (66.7%)
Local HTA evidence is mandatory in decision-making	0 (0.0%)	10 (33.3%)
**(b) Legislation on organizational structure for HTA appraisal**		
There is no public committee or institute for the appraisal process	20 (64.5%)	0 (0.0%)
A committee is appointed for the appraisal process	6 (19.4%)	0 (0.0%)
The committee is appointed for the appraisal process with the support of academic centers and independent expert groups	2 (6.5%)	5 (16.7%)
A public HTA institute or agency is established to conduct a formal appraisal of HTA reports or submissions	2 (6.5%)	4 (13.3%)
Public HTA institute or agency is established to conduct a formal appraisal of HTA reports or submissions with the support of academic centers and independent expert groups	0 (0.0%)	14 (46.7%)
Several public HTA bodies are established without central coordination of their activities	1 (3.2%)	0 (0.0%)
Several public HTA bodies are established with central coordination of their activities	0 (0.0%)	7 (23.3%)
**4. Scope of HTA Implementation**		
**(a) Scope of technologies (multiple choice)**		
HTA is not applied to any health technologies	16 (51.6%)	0 (0.0%)
Pharmaceutical products	15 (48.4%)	26 (83.9%)
Medical devices	2 (6.5%)	27 (87.1%)
Prevention programs and technologies	1 (3.2%)	26 (83.9%)
Surgical interventions	1 (3.2%)	23 (74.2%)
Other scope of technologies (separated by commas)	0 (0.0%)	0 (0.0%)
**(b) Depth of HTA use in pricing and/or reimbursement decision of health technologies**		
HTA is not applied to any health technologies	15 (48.4%)	0 (0.0%)
Only new technologies with significant budget impact	11 (35.5%)	2 (6.5%)
Only new technologies	3 (9.7%)	6 (19.4%)
New technologies + revision of previous pricing and reimbursement decisions	2 (6.5%)	23 (74.2%)
**5. Decision criteria**		
**(a) Decision categories (multiple choice)**		
None of the below categories are applied	7 (22.6%)	0 (0.0%)
Unmet medical need	4 (12.9%)	17 (54.8%)
Healthcare priority	5 (16.1%)	27 (87.1%)
Assessment of therapeutic value	5 (16.1%)	26 (83.9%)
Cost-effectiveness	16 (51.6%)	22 (71.0%)
Budget impact	18 (58.1%)	17 (54.8%)
Other decision categories	0 (0.0%)	1 (3.2%)
**(b) Decision thresholds**		
Thresholds are not applied	24 (77.4%)	0 (0.0%)
Implicit thresholds are preferred	4 (12.9%)	6 (20.0%)
Explicit soft thresholds are applied in decisions	2 (6.5%)	20 (66.7%)
Explicit hard thresholds are applied in decisions	1 (3.2%)	4 (13.3%)
**(c) Multi-criteria decision analysis**		
No explicit multi criteria decision framework is applied	12 (92.3%)	2 (7.4%)
Explicit multi criteria decision framework is applied	1 (7.7%)	25 (92.6%)
**6. Quality and transparency of HTA implementation**		
**(a) Quality elements of HTA implementation (multiple choice)**		
None of the below quality elements are applied	22 (71.0%)	0 (0.0%)
Published methodological guidelines for HTA/economic evaluation	9 (29.0%)	20 (64.5%)
Regular follow-up research on HTA recommendations	1 (3.2%)	19 (61.3%)
A checklist to conduct a formal appraisal of HTA reports or submissions exists but not available for public	2 (6.5%)	8 (25.8%)
A published checklist is applied to conduct a formal appraisal of HTA reports or submissions	0 (0.0%)	23 (74.2%)
**(b) Transparency of HTA in policy decisions**		
Technology assessment reports, critical appraisal and HTA recommendation are not published	27 (87.1%)	0 (0.0%)
HTA recommendation is published without details of technology assessment reports and critical appraisal	1 (3.2%)	10 (32.3%)
Transparent technology assessment reports, critical appraisals and HTA recommendations	3 (9.7%)	21 (67.7%)
**(c) Timeliness**		
HTA submission and issuing recommendation have no transparent timelines	24 (85.7%)	1 (3.4%)
HTA submissions are accepted/conducted following a transparent calendar, but issuing recommendation has no transparent timelines	3 (10.7%)	4 (13.8%)
HTA submissions are accepted continuously and issuing recommendation has transparent timelines	1 (3.6%)	24 (82.8%)
**7. Use of local data**		
**(a) Requirement of using local data in technology assessment**		
No mandate to use local data	22 (73.3%)	0 (0.0%)
The mandate of using local data in certain categories without the need for assessing the transferability of international evidence	7 (23.3%)	2 (6.7%)
The mandate of using local data in certain categories with the need for assessing the transferability of international evidence	1 (3.3%)	28 (93.3%)
**(b) Access and availability of local data**		
Limited availability or accessibility to local real-world data	26 (86.7%)	0 (0.0%)
Up-to-date patient registries are available in certain disease areas, but payers' databases are not accessible for HTA doers	4 (13.3%)	2 (6.7%)
Payers' databases are accessible for HTA doers, patient registries are not available or accessible in the majority of disease areas	0 (0.0%)	6 (20.0%)
Up-to-date patient registries are available in certain disease areas and payers' databases are accessible for HTA doers	0 (0.0%)	22 (73.3%)
**8. International collaboration**		
**(a) international collaboration, joint work on HTA (joint assessment reports) and national/regional adaptation (reuse) (multiple choice)**		
No involvement in joint work; and no reuse of joint work or national/regional HTA documents from other countries	27 (90.0%)	0 (0.0%)
Active involvement in joint work (e.g., EUnet HTA Rapid REA, full Core HTA)	1 (3.3%)	15 (50.0%)
National/regional adaptation (reuse) of joint HTA documents	1 (3.3%)	15 (50.0%)
National/regional adaptation (reuse) of national/regional work performed by other HTA bodies in other countries	1 (3.3%)	15 (50.0%)
**(b) International HTA courses for continuous education on HTA**		
Limited interest in (1) developing / implementing of and (2) participating at international HTA courses	23 (74.2%)	0 (0.0%)
Interest only in regular participation at international HTA courses	8 (25.8%)	4 (12.9%)
High interest in (1) developing / implementing of and (2) participating at international HTA courses	0 (0.0%)	27 (87.1%)
For each question, each expert chose 1 of the available options for the current status and 1 of the options for preferred status. e.g., for question 1a: *an expert chose “No training” in the current status and “Permanent graduate program with short courses” for the preferred status, this means he thinks there are currently no training programs and he would prefer that in 10 years, there will be permanent graduate programs with short courses*		

**Table 2:** Draft recommendations based on major gaps between the current and preferred status of HTA implementation according to the eight domains of the scorecard survey.

**Domain**	**Recommendations**
Capacity-building	More postgraduate HTA programs are recommended based on country-specific needs.
HTA funding	Public funding should be increased for both HTA research and critical appraisal, as well as increasing the private budget through submission fees to reach balanced funding for critical appraisals.
Legislation on HTA	Establishment of multiple HTA bodies within a country preferably with central coordination.
Scope of HTA implementation	Extending the scope of HTA from pharmaceuticals to non-pharmaceuticals is recommended in addition to revising previous policy decisions on top of evaluating new healthcare technologies.
Decision criteria	For cost-effectiveness, explicit soft thresholds should be used. In addition, several other criteria other than cost-effectiveness and budget impact have to be considered by applying multi-criteria decision analysis (MCDA).
Quality and transparency of HTA implementation	Publish and use methodological guidelines and checklists for critical appraisal is recommended to improve HTA work quality. In addition, the deliverables and timeliness of the HTA processes have to be published in the public domain.
Use of local data	Developing more patient registries and utilizing local claims data is recommended with the availability of an electronic payer's database.
International collaboration	Organizing and participating in international HTA courses is highly recommended as well as working on and adapting joint HTA.

The survey results indicate the urgent need to expand basic and advanced educational programs on HTA in Egypt to build a more effective HTA system. Additionally, the findings indicate that locally collected evidence should receive higher priority in policy decisions.

#### Expert interviews

Ten key expert interviews were conducted to assess the feasibility of the findings derived from the preferred HTA status survey in Egypt and to provide details about the implementation timelines, as well as steps and barriers that might arise while implementing the recommendations. Interviewees represented international organizations, the pharmaceutical industry, the government sector, and academic institutions. The governmental organizations included the Health Insurance Organization (HIO), Ministry of Health and Population (MoHP), UHIA, UPA, and Egyptian Drug Authority (EDA).

### HTA domains specific results

The proposed roadmap and recommendations for specific actions based on the HTA scorecard and interviews are summarized below. The results are broken down into eight HTA domains based on the HTA scorecard.

#### HTA capacity-building

All interviewees concurred with the HTA roadmap survey results and recommended more postgraduate HTA programmes based on country-specific needs. Half of them opined that training should focus not only on theoretical knowledge but also on hands-on training. Three indicated that institutional-based capacity-building is the most appropriate approach. Two interviewees recommended that health economics should be included in undergraduate training. One stressed the need to develop capacities in different specialties, including epidemiology and biostatistics, not just health economics.

Several interviewees (*n* = 5) suggested timelines for capacity development. They suggested that in the short term, there should be more courses on developing technical skills for those with a sound theoretical background; for example, economic modeling skills could be developed through three-month to one-year crash courses and diplomas. Within 3–5 years, another master's degree in health economics—other than the one at Cairo University—with a more technical orientation should be launched. Additionally, health economics should be more widely integrated into undergraduate training. Finally, in the long term (within 6–10 years), a doctorate program should be initiated to expand the base of HTA experts who can potentially establish and lead educational courses. In general, within 5e years, Egypt should have a wide pool of capacities with essential multidisciplinary skills in HTA, with special focus on basic and applied health economics.

#### Funding (HTA assessment and critical appraisal)

There are two main components of HTA, the assessment and appraisal. HTA assessment is usually performed by the marketing authorization holder. The submitted dossier includes evidence related to the target patient population, relative efficacy, economic evaluation, cost-effectiveness and other related research. On the other hand, critical appraisal involves validating the submitted evidence in the HTA assessment, then evaluating it in light of several factors such as budget constraints and local health policies, to construct a recommendation for decision makers (29). Experts were asked how each of the two HTA components should be funded.

Although survey respondents equally preferred funding of the critical appraisal from submission fees or public resources, interviewees agreed on private funding through submission fees is the most reasonable option for the next decade similarly to the pharmaceutical regulatory system, where the review of new drug applications is funded from the regulatory fees paid by pharmaceutical companies.

Two experts raised a flag that bias might occur in favor of the manufacturer due to the influence of the submission fees. Notably, they suggested that the submission fees should be reasonable without a profit margin. Interviewees highlighted that public funding may never be the dominant funding model for critical appraisals in Egypt, although partial public funding will be necessary especially in those areas where HTA is initiated by public or academic institutes.

Seventy-one percent of the survey respondents pointed that sufficient public funding for HTA research with supportive private funding is expected in the future and 23% preferred HTA research to be dominantly funded from public resources. While interviewees considered that public funding should have a role, they agreed that it would not be realistic to discourage investment of the private sector to HTA research in the forthcoming years with expectedly limited public resources in all health care sectors. Pharmaceutical companies make huge global investments into preparing HTA dossiers and health economic models in many different countries, and it makes no sense to ignore these research outputs and rely only or dominantly on local public funding in HTA research. As an example, development of local health economic models from scratch by public stakeholders in Egypt would be far more expensive than encouraging the local adaptation of global health economic models by pharmaceutical companies. One interviewee even said, “HTA research will never be dominantly publicly funded in Egypt”.

#### Legislation on HTA

All survey respondents agreed that either (1) international or local HTA evidence, or (2) strictly local evidence, must be considered in decision-making. Seven interviewees favored having a central coordination or a central HTA agency rather than multiple HTA agencies - they flagged potential overlap of functions, leading to biased results if there was no central coordination. One interviewee proposed that the HTA agency should be an independent government body; others proposed multiple HTA bodies. One interviewee emphasized that HTA must be obligatory and should be used for pricing, replacing the current methodology of external price reference.

Interviewees recommended that HTA should be obligatory by law for pricing and reimbursement for high budget impact innovative technologies. Additionally, they recommended the establishment of HTA units at the UPA, and UHIA. On the midterm (next 3–5 years), central coordination of HTA activities between the different HTA bodies should be established by law, preferably mandated by the parliament. On the long term (5–10 years), there should be a decree to merge HTA units from different governmental organizations when feasible.

#### Scope of HTA implementation

There was consensus regarding the impracticability of assessing all types of technologies initially—a gradual assessment mechanism was preferred. Six interviewees prioritized the assessment of innovative pharmaceuticals with significant budget impact and suggested broadening the scope after the first 2 years; two interviewees recommended commencing with both innovative pharmaceuticals and medical devices with significant budget impact, and then expanding the scope. Two interviewees suggested beginning with medical devices and then expanding the scope to pharmaceuticals—however, the challenge will be the scarcity of data and shortage of experts in medical device HTA. One of the two interviewees remarked, “It would be optimal to start with medical devices, but it might not be feasible.” Most of the interviewees (*n* = 9) recommended expanding the types of health technologies to be assessed within 3–5 years. The establishment of HTA for all health technologies, including services, in the long run was recommended by all interviewees. Regarding the revision of previous decisions, three interviewees expected revisions to begin within 3–5 years. Six interviewees expected it to occur at least after 6 years—one of them said, “Revision is farfetched maybe not in 10 years even,” and another expected the full scope of HTA to be implemented in 10–15 years.

#### Decision criteria

All interviewees agreed that cost-effectiveness and budget impact analyses should be used as decision criteria for HTA. Many of them highlighted the significance of considering other criteria such as cost-effectiveness threshold details and using scoring systems, such as MCDA, to choose among alternatives and manage entry agreements for reimbursement.

Interviewees' emphasis was on the cost-effectiveness threshold, and opinions were split between implementing an explicit or implicit threshold. One interviewee mentioned potential impediments in the implementation of an explicit threshold; two interviewees sounded the alarm that manufacturers would price their products based on the threshold. Four interviewees suggested the use of multiple thresholds: one of them proposed using multiple thresholds based on disease area or severity, and another proposed different thresholds—one for market authorization price and another for reimbursement. Two interviewees proposed using a hard threshold, and four proposed using a soft threshold allowing for further negotiation or managed entry agreements (MEAs).

Most of the interviewees (*n* = 7) emphasized the importance of MCDA. It has been mentioned that MCDA is necessary for tenders, especially for off-patent pharmaceuticals. One of them highlighted the price weight in the MCDA, stating the need to put a reasonable weight on the price.

#### Quality and transparency of HTA implementation

All experts agreed to implement both the guidelines and specific timelines for the HTA process. However, two of them questioned the pragmatism of publishing timelines for the HTA appraisal process—from submission to issuing recommendations. Three interviewees indicated the impracticability of implementing such guidelines in the short term, suggesting it would take 3–5 years for full implementation. One mentioned that within 1–2 years, we could have deliverables and timelines for the process, and within 3–5 years, we could have published guidelines for economic evaluation.

#### Use of local data

Most interviewees (*n* = 7) acknowledged the importance of local data use in HTA, but many (*n* = 5) highlighted existing hurdles such as scarcity and accessibility of local data and absence of a clear legal framework for data requests. Additionally, mass patient records in Egypt are usually secured, with restricted accessibility. Therefore, even if the data are available, it is sometimes not approved for sharing or publishing. One expert said, “The problem is not with data availability as much as the possibility to utilize.”

For this, a legal framework for data sharing should be established. The information circulation law should provide advice on what data are allowed to be exchanged, and the required fees. Furthermore, data may be provided as summary statistics, not raw data, to decrease the sensitivity. Regarding data availability, although the current quality of available data might not be optimal, the General Authority for Healthcare and UHIA will have useful data because of the ongoing automation. It is anticipated that the data will be readily utilized in 3–5 years.

#### International collaboration

All interviewees supported international collaboration. Several bodies have been proposed for collaboration, including the National Institute for Health and Care Excellence from the UK; the French National Authority for Health; and HTA bodies in Southeast Asia, South Korea, Taiwan, and Italy. One interviewee emphasized formal collaboration with regional HTA bodies for hands-on training and sharing experiences.

### Summary of recommendations for specific actions

The results are summarized and presented in Table 3 according to the key elements of HTA implementation, as stated above. The recommendations in the table were based on the interviewees' validation of the survey results.

**Table 3:** HTA implementation in Egypt recommendations for specific actions.

**Element**	**Action 1–2 Years**	**3–5 Years**	**6–10 Years**
Capacity-building	• Postgraduate diploma in HTA focused on country specific needs and technical skills (what will be done in routine practice)	• Another master's program more technical oriented • Undergraduate training in Health Economics development	• Train the trainers program • PhD program
HTA funding	• Research works should be powered by manufacturers • Set initial submission fees for appraisal	• Redefine submission fees based on HTA department cost calculation.	
Legislation on HTA	• HTA is obligatory for pricing, and reimbursement of selected technologies • Empower the HTA unit in the Egyptian Drug Authority for the purpose of out-of-pocket pricing • Establish a supplementary HTA unit in the Health Insurance Organization (HIO) for the purpose of assessing the budget impact in the organization • Establish a supplementary HTA unit in the Unified Procurement Authority (UPA) • Engage in managed entry agreements with manufacturers	• Establish a supplementary HTA unit within the Universal Health Insurance Authority (UHIA) for the purpose of assessing the budget impact in the organization • Central coordination of HTA activities between the different HTA bodies to avoid duplication of work	• Merge the HTA units from different governmental organizations.
Scope of HTA implementation	• Start assessing innovative pharmaceuticals with high budget impact to support reimbursement decisions	• Expand the scope of HTA to cover health programs and medical devices, as well as diagnostics and new interventional therapies for the purpose of reimbursement • Expand the use of HTA for the purpose of OOP pricing	• full scope of HTA or go with full HTA implementation covering all technologies, including services • revision of previous decisions for reimbursement and OOP pricing
Decision criteria	• Publishing explicit multiple thresholds for out-of-pocket pricing and reimbursement. Also, differential/multiple thresholds based on the severity of disease. • Implement systematic scientific MCDA in tenders for OOP as well as medical devices in different organizations • Pilot managed entry agreements MEAs cases	• The use of systematic, scientific MCDA is obligatory for all tenders.	• State MEAs as obligatory for reimbursement.
Quality and transparency of HTA implementation		• Publishing guidelines for the HTA process. • Publishing timelines for the HTA process.	
Use of local data	• Several costing studies for different disease areas.	• Readily use of local data in HTA. Primarily cost data and can extend to real world clinical evidence occasionally.	• National public data bank for input parameters for HTA. • The information circulation law should provide advice on what data is allowed to be exchanged and the fees.
International collaboration	• International partnership in training programs	• Partial adaptation of the work of international HTA bodies or organizations	• Establishing an international HTA network for collaboration on transferable HTA component with countries with similar economic status (Middle income countries) and healthcare structure (Recent implementation of UHC).

## Discussion

HTA implementation in Egypt has significantly progressed; however, aspirations are still higher. The financing structure under the new healthcare system reform has led to the centralization of decision-making. Consequently, the burden on the government—as well as its control—has increased (9), which increases the suitability of conducting HTA at the central level (30).

Given the current Egyptian healthcare system structure, the country is facing systemic inequalities and inefficiencies that have limited the effectiveness of its health system. The government is expected to have an extremely high negotiation power in the near future under the new universal insurance, because of the extended beneficiary coverage and expanded benefit package. This necessitates efficient resource allocation to maximize benefits. HTA can play a significant role in achieving healthcare financing objectives by scientifically and systematically assessing health technologies as well as pricing and reimbursement policies.

There are several barriers to HTA implementation, considering the current Egyptian healthcare system structure, as discussed earlier. Nevertheless, stakeholders can identify the potential of HTA implementation in enhancing the healthcare system in Egypt, especially amid the healthcare reform. The positive expectations of decision-makers are evident from the existing progress and initiatives taken toward HTA implementation in Egypt over the past decade.

As in many countries in the MENA region, steps were taken to implement HTA in Egypt; nevertheless, much has to be done before reaping full and fruitful benefits. Thus, a roadmap is necessary to guide decision-makers based on previous experiences and the viewpoints of experts. The results of the roadmap and associated recommendations for specific actions provide clear steps for establishing a successful HTA structure and its related activities.

Here, we propose a clear roadmap with recommendations for specific actions to achieve full HTA implementation in Egypt, based on expert surveys and interviews.

There are certain fundamental components of the roadmap. One of them is gradual capacity-building to guarantee sufficiency of experts. HTA capacity-building in Egypt has advanced over the past decade. However, more postgraduate programs are required based on country-specific needs.

HTA appraisal and research should be funded mainly by manufacturers—supplemented by some public funding in research areas initiated by public or academic institutes. The funding by the public sector is currently limited; thus, more HTA funding needs to be secured through both public and private funding. The allocation of public resources to HTA indicates political support, which should facilitate the implementation process (31). It was recommended that the submission fees paid by manufacturers for the appraisal of their models should be low. This recommendation mainly came from interviewees with a governmental background, to prevent any bias due to the high submission fees paid.

As in many other countries, HTA in Egypt should begin with a focus on innovative pharmaceuticals, especially those with a high budget impact. In the future, more efforts are needed to expand to additional technologies within the scope of HTA and revise previous policy decisions. Several criteria other than cost-effectiveness and budget impact analyses, such as unmet medical needs and healthcare priority, should be considered by decision-makers to improve HTA implementation and apply explicit willingness-to-pay thresholds. Most stakeholders recommend using multiple and soft cost-effectiveness thresholds. Applying an explicit MCDA framework, particularly tenders, is essential for consistent decisions. To enhance transparency, HTA deliverables and timelines must be published.

Furthermore, the system should benefit from transferable international evidence (e.g., relative effectiveness) and rely on local data, where international evidence is not transferable (e.g., cost analysis). Finally, international collaboration with prestigious organizations, enhancing local political will, and adopting the best international and regional practices shall help empower HTA bodies in Egypt. Egypt's HTA bodies should participate in joint HTA documents and courses with international bodies. In the long run, establishing a central HTA body is fundamental to avoiding duplication of efforts.

A problem that was highlighted is the gap between HTA research and actual reimbursement decision-making. Decision-makers need to actively implement HTA. Publishing guidelines and checklists for critical appraisal is recommended to enhance the comparability of studies and improve their quality.

The same HTA scorecard was previously applied to the MENA region (31), and we compared the results from Egypt with those from the MENA region. The comparison revealed that there is consensus on some major elements of HTA implementation and some heterogeneity in other aspects.

The MENA survey outcomes revealed the limited availability of HTA training options and that launching graduate and postgraduate programs in the future is preferred. Moreover, currently, HTA has no formal role in decision-making. However, almost all respondents preferred to see formal HTA processes and use local data in the future. HTA is recommended in pharmaceuticals, medical devices, and prevention programs. Almost all respondents believed that joining international bodies for collaborative purposes would be helpful for both decision-making and capacity-building.

Compared to Egypt's survey results, both survey results are quite similar in terms of the preferred structure of HTA in almost all domains, except for a few elements, such as funding. Unlike in the MENA region, experts in Egypt prefer a dominant private funding model for HTA research and appraisal, which might be attributed to the lower gross domestic product per capita in Egypt compared to the sample in the MENA region survey (31). Egyptian experts put less emphasis on future budgetary impacts compared to experts in other regional countries. In contrast to the MENA region, experts in Egypt prefer to publish recommendations without details of technology assessment reports and critical appraisals; experts in the MENA region recommend more transparency by publishing these details. A scorecard comparing the preferred status results from Egypt with the MENA region is presented in Supplementary Table 2.

### Recommendations

Based on the survey results, interviews, and discussions, a complete list of recommended actions and a clear roadmap was created for the short-term (next 1–2 years), mid-term (3–5 years), and long-term (6–10 years); the recommended actions are summarized in Table 3. According to the experts interviewed, if these actions are executed within the proposed timelines, HTA would be fully implemented and functional in Egypt.

### Limitations

We cannot claim that the sample in either the survey or the interviews are fully generalizable or representative, but this is mostly because of limitation of funding and resources. Furthermore, it is seldom possible to find stakeholders from different entities with knowledge of HTA who can address these questions. However, we did our best to include various stakeholders that represent multiple key entities. The HTA scorecard results from 2018 might be considered outdated; however, the most crucial element in the survey is the aspired situation, which is not expected to significantly change over this time period as opposed to the current status. Furthermore, later interviews validated the scorecard recommendations. Since this research commenced, several steps have been taken toward HTA implementation, and a few proposed actions may have already been implemented at the time of publication of this study.

## Author contributions

AF and ZK designed this study. MGa, AS, AA, MGe, MAn, MAm, AK, AE, AH, SAbd, HE, NF, RA, and HD responded to the interviews and provided input. SAba, AF, RA, and HD managed the data-collection phase. AF and BE drafted the manuscript. All the authors participated in the proposal of specific actions and recommendations, revised the manuscript, provided comments, and approved the final version of the manuscript.
