# Supplementary material for: A roadmap toward implementing health technology assessment in Egypt
Source: Front Public Health. 2022 Dec 13;10:896175. doi: 10.3389/fpubh.2022.896175 (PMC9792961; doi:10.3389/fpubh.2022.896175)
Supplement: Supplementary file 1 [file Table_1.DOCX]

**Demographic characteristics of the survey respondents**

| **Question** | **No. of answers** |
| --- | --- |
| **Main employment** |  |
| Public sector | 26 (83.9%) |
| Private sector | 5 (16.1%) |
| Main employment - Public sector |  |
| **Major training** |  |
| Pharmacy | 15 (48.4%) |
| Medicine | 7 (22.6%) |
| Economics | 0 (0.0%) |
| Multidisciplinary (at least two master degrees from the above list) | 8 (25.8%) |
| Other | 1 (3.2%) |
| **Age** |  |
| Below 30 | 3 (9.7%) |
| Between 30 and 50 | 23 (74.2%) |
| Above 50 | 5 (16.1%) |
