# Supplementary material for: A roadmap toward implementing health technology assessment in Egypt
Source: Front Public Health. 2022 Dec 13;10:896175. doi: 10.3389/fpubh.2022.896175 (PMC9792961; doi:10.3389/fpubh.2022.896175)
Supplement: Supplementary file 2 [file Table_2.DOCX]

**Comparison Egypt and Middle East HTA Roadmap**

| **Region/Country** | **Egypt** | **Middle East** |
| --- | --- | --- |
| **Question** | **Preferred** | **Preferred** |
| **HTA Capacity Building** |  |  |
| **Education** |  |  |
| No training | 0 (0.0%) | 2 (4.0%) |
| Project based training and short courses | 3 (9.7%) | 2 (4.0%) |
| Permanent graduate program with short courses | 4 (12.9%) | 8 (16.0%) |
| Permanent graduate and postgraduate program with short courses | 24 (77.4%) | 39 (76.0%) |
| **HTA Funding** |  |  |
| **Financing critical appraisal of technology assessment** |  |  |
| No funding for critical appraisal of technology assessment reports or submissions | 1 (3.3%) | 4 (7.8%) |
| Dominantly private funding (e.g., submission fees) by manufacturers for the critical appraisal of technology assessment reports or submissions | **14 (46.7%)** | **12 (21.6%)** |
| Dominantly public funding for critical appraisal of technology assessment reports or submissions | 15 (50.0%) | 37 (70.6%) |
| **Financing health technology assessment (i.e., HTA research)** |  |  |
| No public funding for technology assessment; private funding is not needed or expected | 0 (0.0%) | 5 (9.8%) |
| No or marginal public funding for research in HTA; private funding is expected | 2 (6.5%) | 7 (11.8%) |
| Sufficient public funding for research in HTA; private funding is also expected | **22 (71.0%)** | **20 (39.2%)** |
| HTA research is dominantly funded from public resources | **7 (22.6%)** | **21 (39.2%)** |
| **Legislation on HTA** |  |  |
| Legislation on the role of HTA process and recommendations in decision-making process |  |  |
| No formal role of HTA in decision-making | 0 (0.0%) | 4 (8.7%) |
| Dominantly international HTA evidence is taken into account in decision-making | 0 (0.0%) | 2 (4.3%) |
| International and additionally local HTA evidence is taken into account in decision-making | 20 (66.7%) | 22 (47.8%) |
| Local HTA evidence is mandatory in decision-making | 10 (33.3%) | 19 (39.1%) |
| **Legislation on organizational structure for HTA appraisal** |  |  |
| There is no public committee or institute for the appraisal process | 0 (0.0%) | 5(9.8%) |
| Committee is appointed for the appraisal process | 0 (0.0%) | 2(3.9%) |
| Committee is appointed for the appraisal process with support of academic centres and independent expert groups | 5 (16.7%) | 3 (5.9%) |
| A public HTA institute or agency is established to conduct formal appraisal of HTA reports or submissions | 4 (13.3%) | 3 (5.9%) |
| Public HTA institute or agency is established to conduct formal appraisal of HTA reports or submissions with support of academic centres and independent expert groups | 14 (46.7%) | 22 (43.1%) |
| Several public HTA bodies are established without central coordination of their activities | 0 (0.0%) | 1 (2.0%) |
| Several public HTA bodies are established with central coordination of their activities | 7 (23.3%) | 17 (29.4%) |
| **Scope of HTA Implementation** |  |  |
| **Scope of technologies** |  |  |
| HTA is not applied to any health technologies | 0 (0.0%) | 4 (4.0%) |
| Pharmaceutical products | 26 (83.9%) | 37 (92.0%) |
| Medical devices | 27 (87.1%) | 37 (78.0%) |
| Prevention programs and technologies | 26 (83.9%) | 34 (66.0%) |
| Surgical interventions | 23 (74.2%) | 34 (64.0%) |
| Other scope of technologies (separated by commas) | 0 (0.0%) | 4 (8.0%) |
| **Depth of HTA use in pricing and/or reimbursement decision of health technologies** |  |  |
| HTA is not applied to any health technologies | 0 (0.0%) | 6 (11.8%) |
| Only new technologies with significant budget impact | 2 (6.5%) | 9 (15.7%) |
| Only new technologies | 6 (19.4%) | 5(9.8%) |
| New technologies + revision of previous pricing and reimbursement decisions | 23 (74.2%) | 34 (62.7%) |
| **Decision criteria** |  |  |
| **Decision categories** |  |  |
| None of the below categories are applied | 0 (0.0%) | 3 (2.0%) |
| Unmet medical need | 17 (54.8%) | 33 (62.7%) |
| Health care priority | 27 (87.1%) | 40 (76.5%) |
| Assessment of therapeutic value | 26 (83.9%) | 40 (78.4%) |
| Cost-effectiveness | 22 (71.0%) | 39 (82.4%) |
| Budget impact | **17 (54.8%)** | **42 (84.3%)** |
| Other decision categories (separated by commas) | 1 (3.2%) | 1 (2.0%) |
| **Decision thresholds** |  |  |
| Thresholds are not applied | 0 (0.0%) | 3 (5.9%) |
| Implicit thresholds are preferred | 6 (20.0%) | 8(15.7%) |
| Explicit soft thresholds are applied in decisions | 20 (66.7%) | 27 (51.0%) |
| Explicit hard thresholds are applied in decisions | 4 (13.3%) | 15 (27.5%) |
| **Multi-criteria decision analysis** |  |  |
| No explicit multi criteria decision framework is applied | 2 (7.4%) | 8 (14.3%) |
| Explicit multi criteria decision framework is applied | 25 (92.6%) | 44 (85.7%) |
| **Quality and transparency of HTA implementation** |  |  |
| **Quality elements of HTA implementation** |  |  |
| None of the below quality elements are applied | 0 (0.0%) | 4 (6.1%) |
| Published methodological guidelines for HTA/economic evaluation | 20 (64.5%) | 24 (53.1%) |
| Regular follow-up research on HTA recommendations | 19 (61.3%) | 23 (44.9%) |
| Checklist to conduct formal appraisal of HTA reports or submissions exists but not available for public | 8 (25.8%) | 19 (36.7%) |
| Published checklist is applied to conduct formal appraisal of HTA reports or submissions | 23 (74.2%) | 34 (67.3%) |
| **Transparency of HTA in policy decisions** |  |  |
| Technology assessment reports, critical appraisal and HTA recommendation are not published | 0 (0.0%) | 3(6.0%) |
| HTA recommendation is published without details of technology assessment reports and critical appraisal | **10 (32.3%)** | **6 (12.0%)** |
| Transparent technology assessment reports, critical appraisals and HTA recommendations | 21 (67.7%) | 44 (82.0%) |
| **Timeliness** |  |  |
| HTA submission and issuing recommendation have no transparent timelines | 1 (3.4%) | 6 (12.0%) |
| HTA submissions are accepted/conducted following a transparent calendar, but issuing recommendation has no transparent timelines | 4 (13.8%) | 5 (10.0%) |
| HTA submissions are accepted continuously and issuing recommendation has transparent timelines | 24 (82.8%) | 42 (78.0%) |
| **Use of local data** |  |  |
| **Requirement of using local data in technology assessment** |  |  |
| No mandate to use local data | 0 (0.0%) | 4 (8.3%) |
| Mandate of using local data in certain categories without need for assessing the transferability of international evidence | 2 (6.7%) | 7 (14.6%) |
| Mandate of using local data in certain categories with need for assessing the transferability of international evidence | 28 (93.3%) | 39 (77.1%) |
| **Access and availability of local data** |  |  |
| Limited availability or accessibility to local real-world data | 0 (0.0%) | 5 (9.8%) |
| Up-to-date patient registries are available in certain disease areas, but payers’ databases are not accessible for HTA doers | 2 (6.7%) | 4 (7.8%) |
| Payers’ databases are accessible for HTA doers, patient registries are not available or accessible in the majority of disease areas | 6 (20.0%) | 6 (11.8%) |
| Up-to-date patient registries are available in certain disease areas and payers’ databases are accessible for HTA doers | 22 (73.3%) | 39 (70.6%) |
| **International collaboration** |  |  |
| International collaboration, joint work on HTA (joint assessment reports) and national/regional adaptation (reuse) |  |  |
| No involvement into joint work; and no reuse of joint work or national/regional HTA documents from other countries | 0 (0.0%) | 2 (4.3%) |
| Active involvement in joint work (e.g., EUnetHTA Rapid REA, full Core HTA) | 15 (50.0%) | 20 (43.5%) |
| National/regional adaptation (reuse) of joint HTA documents | 15 (50.0%) | 27 (56.5%) |
| National/regional adaptation (reuse) of national/regional work performed by other HTA bodies in other countries | 15 (50.0%) | 36 (71.7%) |
| **International HTA courses for continuous education on HTA** |  |  |
| Limited interest in (1) developing / implementing of and (2) participating at international HTA courses | 0 (0.0%) | 6 (11.8%) |
| Interest only in regular participation at international HTA courses | **4 (12.9%)** | **2 (3.9%)** |
| High interest in (1) developing / implementing of and (2) participating at international HTA courses | 27 (87.1%) | 46 (84.3%) |
